# US Veterans Administration Autosomal Dominant Polycystic Kidney Disease Cohort: Demographic, Comorbidity, and Key Laboratory Data Characteristics

**DOI:** 10.34067/KID.0000000000000405

**Published:** 2024-03-01

**Authors:** Julia W. Gallini, Christine L. Jasien, Michal Mrug, Xiangqin Cui

**Affiliations:** 1 Foundation for Atlanta Veterans Education and Research, Decatur, Georgia; 2 Department of Veterans Affairs Medical Center, Atlanta VA Health Care System, Decatur, Georgia; 3 Department of Medicine, University of Alabama at Birmingham, Birmingham, Alabama; 4 Department of Veterans Affairs Medical Center, Birmingham, Alabama; 5 Department of Biostatistics and Bioinformatics, Rollins School of Public Health, Emory University, Atlanta, Georgia

**Keywords:** ADPKD, CKD, cystic kidney, dialysis, ESKD

## Abstract

**Key Points:**

- We built a cohort of 12,217 patients diagnosed with autosomal dominant polycystic kidney disease from 1999 to 2020 in the national Veteran Affairs electronic medical record system.
- We characterized the cohort on demographics, comorbidities, and key laboratory measurements.

**Background:**

We used the largest integrated US healthcare system, the Veterans Health Administration, to establish a robust resource for demographic, longitudinal outcome, and predictive modeling studies in autosomal dominant polycystic kidney disease (ADPKD).

**Methods:**

We built the ADPKD cohort by extracting the relevant electronic health record data from nationwide Veterans Health Administration database (years 1999–2020).

**Results:**

We identified 12,217 patients diagnosed with ADPKD. By the end of the 20-year study period, 5342 patients with ADPKD were deceased, 1583 were alive but reached ESKD, and 4827 remained alive without ESKD. Most demographic characteristics of this ADPKD cohort resemble the total US veteran population. For example, 94% were male patients, 45% age 65 years or older, 85% non-Hispanic, and 66% white; however, 19% were Black/African Americans (versus 12% in the general veteran population; a relevant enrichment after considering age and sex distributions between races). The comorbidities overrepresented in the ADPKD cohort include hypertension (89% versus 50%), diabetes (32% versus 22%), depression (40% versus 10%), chronic obstructive pulmonary disease (30% versus 6%), and congestive heart failure (21% versus 1%). By contrast, obesity was underrepresented in veterans with ADPKD (30% versus 41%).

**Conclusions:**

We established a large electronic medical record-based cohort of ADPKD veterans. Here, we provide initial analysis of its demographic, comorbidity, and key laboratory data.

## Visual Abstract

**Figure:**
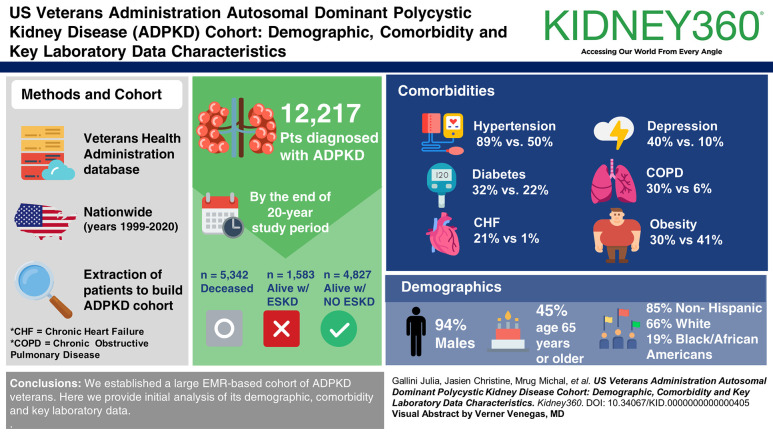

## Introduction

Autosomal dominant polycystic kidney disease (ADPKD) is the fourth leading cause of ESKD. However, ADPKD can also manifest with a spectrum of additional potentially serious renal and extrarenal manifestations.^1^

The critical insight into the clinical relevance of ADPKD manifestations, their progression, staging, and management mainly stem from studies of well-characterized focused cohorts of patients with ADPKD.^2–6^ They include the Consortium of Radiologic Imaging Study of Polycystic Kidney Disease study (241 patients with ADPKD), the most extensive observational multicenter ADPKD study.^2^ Another outstanding data source is the Halt Progression of Polycystic Kidney Disease study (1044 ADPKD patients), the first large multicenter interventional trial in ADPKD that tested effects of renin-angiotensin-aldosterone system blockade on structural kidney end point in early disease (study A) and more conventional kidney functional end point in the late disease (study B). Among additional significant sources of ADPKD clinical data are ADPKD patient cohorts that were established at local medical centers or regional medical systems. They include the Mayo Clinic cohort (590 patients with ADPKD),^5^ the South Korea cohort (364 patients with ADPKD; *e.g*., used to analyze the correlation of kidney and liver volume and kidney function),^6^ and the Genkyst cohort (2449 patients with ADPKD used to examine the relationship between genotype and phenotype in an ADPKD population).^4^ To enable analyses of larger expanded ADPKD datasets, approximately seven decades of patient visits from 2498 patients in observational ADPKD registries at the University of Colorado—Denver, Mayo Clinic, and Emory University were standardized and aggregated into the Polycystic Kidney Disease Outcomes Consortium database using a Clinical Data Interchange Standards Consortium Standard Data Tabulation Model structure.^7^

An alternative to ADPKD-centered cohorts from registries and clinical trials that can capture higher numbers of patients with ADPKD is based on the extraction of ADPKD relevant information from large registries or clinically relevant databases that are not strictly focused on ADPKD. Such analyses might show the data in a more representative, less biased context. Among the first examples of such an ADPKD, data extraction is the use of the European Renal Association-European Dialysis and Transplant Association Registry (20,596 ADPKD patients) to evaluate the effects of conventional treatment on the need for RRT in patients with ADPKD.^8^ Similarly, ADPKD relevant data were extracted from the United States Renal Data System (USRDS) (23,647 ADPKD patients^9^) to examine racial differences in ESKD due to ADPKD. However, in the case of both the European Renal Association-European Dialysis and Transplant Association and USRDS registries, the data collected are still primarily focused on a specific outcome—the ESKD. Therefore, the utility of these resources for studying other renal and extrarenal ADPKD manifestations is limited.

Perhaps the most inclusive and least biased ADPKD relevant data collection can be achieved by clinical data extraction from electronic health records (EHRs) of large healthcare organizations. One of the examples of this research direction is the EHR data extraction that was recently used to characterize ADPKD prevalence among the racially diverse US population as reflected by the participants in the Kaiser Permanente Southern California (KPSC) health system (9,071,375 KPSC members between 2002 and 2018, of whom 3868 were diagnosed ADPKD).^10^ Interestingly, this study revealed the highest ADPKD prevalence among Black patients with ADPKD compared with any other race group.

In this study, we extracted and characterized ADPKD relevant data to create the Veteran Affairs (VA)-ADPKD cohort from the largest integrated healthcare system in the United States, the Veterans Health Administration (VHA). The VHA currently serves approximately nine million enrolled veterans in 1293 healthcare facilities each year. The VHA's EHR system (VA Corporate Data Warehouse [CDW]) provides access to the medical records of veterans across the United States, reaching back to October 1999 with more than 24 million patients. Therefore, the VA CDW is an attractive resource for establishing a large ADPKD cohort for future outcome studies and for future longitudinal analyses.

## Methods

We extracted EHR data from the Department of VA CDW. Patients were included in the cohort if they were assigned relevant diagnostic codes between January 10, 1999, and January 2, 2020, defined by the ninth and tenth revision of the International Statistical Classification of Diseases and related health problems (ICD-9 and ICD-10). Under the ICD-9 code hierarchy, code 753.12 (polycystic kidney disease, unspecified) branches to 753.13 (ADPKD) and 753.14 (polycystic kidney-autosomal recessive; ARPKD). Since the likelihood of an ARPKD diagnosis in the US veteran population is extremely low in the context of the ARPKD clinical manifestations profile,^11^ we assumed that in most cases, the ARPKD diagnostic code was used in error instead of the ADPKD code (even if it was applied correctly, the impact on reported outcomes would be low since combined ICD-9 and ICD-10 codes for ARPKD comprised only 1% of the total cohort). Therefore, we extracted the data from CDW for all three ICD-9 codes. Similarly, we extracted the data for the corresponding ICD-10 codes (implemented in 2015). They include Q61.2 (polycystic kidney, adult type) applicable for ADPKD and Q61.19 (other polycystic kidney, infantile type) for ARPKD. However, to limit inclusion of non-ADPKD patients (and at the cost of excluding some patients with ADPKD), we omitted the code Q61.3 (polycystic kidney, unspecified) that is defined more broadly as congenital cystic kidney disease and multiple congenital cysts of kidney. We opted for such a more stringent approach that may have excluded some patients with ADPKD to improve the proportion of patients with correctly diagnosed ADPKD in our cohort. We initially used this cohort for analyses centered on coronavirus disease 2019 outcomes among veterans with ADPKD.^12^ By contrast, the current manuscript is focused on a more systematic assessment of ADPKD relevant demographic and clinical data (see below). This study was approved by the Emory University Institutional Review Board (IRB00115069).

Demographic data for the VA cohort (*e.g*., race, ethnicity, sex, and age) were extracted from the VA CDW; frequencies and percentages were calculated. Age was defined as the patient age when the ADPKD diagnosis code first appeared in the records, which is often later than the first patient visit in the VHA system. Age and sex distributions within each race were calculated.

Key ADPKD outcomes of interest were extracted, including ESKD diagnosis, transplant, dialysis use, and death. Patients were divided into three main categories: alive with no ESKD diagnosis, alive with ESKD, and deceased. In addition, we extracted data related to chronic diseases to determine the effects of common comorbidities on ADPKD outcomes. The composite effect of comorbidities was assessed using a weighted Elixhauser index based on 29 conditions for in-hospital mortality: HIV/AIDS, alcohol abuse, iron deficiency anemia, rheumatoid arthritis/collagen vascular diseases, chronic blood loss anemia, congestive heart failure, chronic obstructive pulmonary disease (COPD), coagulopathy, depression, diabetes, drug abuse, hypertension, hypothyroidism, liver disease, lymphoma, fluid and electrolyte disorders, metastatic cancer, other neurologic disorders, obesity, paralysis, peripheral vascular disorders, psychoses, pulmonary circulation disorders, renal failure, solid tumor without metastasis, peptic ulcer disease excluding bleeding, valvular disease, and weight loss.^13^ Additional comorbidities studied included intracranial aneurysm, bronchiectasia, intracranial bleeding, cystic liver/pancreas, infertility, nephrolithiasis, pain, and seminal vesical cysts. Patients were defined as having/not having each comorbidity based on ICD codes. Frequencies and percentages were calculated for each comorbidity overall and by race. Odds ratios (ORs) were calculated to compare the prevalence of comorbidities between Black/African American patients and White patients. The median and interquartile range (IQR) of the weighted in-hospital mortality Elixhauser Index^13^ were also calculated for the whole cohort and by race.

Laboratory measurements of interest were also extracted from VA CDW. Serum laboratory test results extracted included creatinine, hemoglobin, white blood cell count, potassium, carbon dioxide, alanine aminotransferase/serum glutamate-pyruvate transaminase, aspartate aminotransferase/serum glutamate-oxaloacetate transaminase, albumin, alkaline phosphatase, uric acid, protein, glucose, phosphorus, calcium, and BUN. Urine laboratory test results included protein-to-creatinine ratio and albumin-to-creatinine ratio. Additional extracted laboratory values included body mass index, body surface area, and mean arterial pressure. Laboratory results determined to be outside of a plausible range (Supplemental Table 1) were deleted to minimize data errors. For each laboratory test, the following descriptive statistics were calculated: a number of cohort members with at least one measurement in the study period, median and IQR of within patient median laboratory value, median and IQR of the number of laboratory values over the study period per patient among those with at least one laboratory value, and the median and IQR of time in days between subsequent laboratory measurements within the same patient.

We calculated eGFR values from the serum creatinine values extracted from VA CDW using the eGFR-CKD Epidemiology Collaboration formula.^14,15^ Values over 250 were deemed implausible and deleted. Since eGFR is a crucial measure of kidney function, we then implemented a thorough data cleaning process to ensure its accuracy. All eGFR values that were calculated within VA CDW at the time of the laboratory draw (eGFR-EHR) had the limitation that the eGFR formula used for the calculation changed multiple times over the study period with no way to trace which formula was used when. Therefore, we first recalculated eGFR using the CKD Epidemiology Collaboration formula and plotted these values against the eGFR-EHR. Several areas of concern were identified by manual backtracking of suspicious data. They included instances of incorrect serum creatinine values due to an incorrect unit entry, the entry of a reciprocal creatinine, or reports of an unrelated test under the creatinine entry. Such incorrect serum creatinine values were corrected or removed from the dataset. This rigorous data cleaning process increased confidence that eGFR values included in the final dataset are accurate and suitable for future analyses (*e.g*., longitudinal modeling).

Key descriptive statistics on eGFR values were plotted using density plots. These statistics included median patient-level eGFR values, patient-level number of eGFR measurements over the study period, patient-level median days between subsequent eGFR measurements, and within-patient range (maximum–minimum) of eGFR values. Statistics were displayed by three groups: patients who died during the study period, patients with ESKD who were alive at the end of the study period, and non-ESKD patients who were alive at the end of the study period.

## Results

Using the 1999–2020 nationwide VA system EHR data, we identified 12,217 patients with ADPKD. This VA-ADPKD cohort is 94% male, 45% older than 65 years, 84% non-Hispanic, and 66% White. These characteristics resemble the entire VA patient population.^16^ The percentage of Black/African American patients in this cohort was 19%, which is higher than the 12% in the total veteran population.

During the 20-year study period, the cohort has 115,707 person years in total with an average of 10.3 years per patient. The cohort has 11,752 patients with vital status information on the basis of the VA death index data and valid eGFR data to confirm ESKD. Among these patients, 45% died (*N*=5342), 14% of were alive but reached ESKD in the VHA system by the end of the study period (*N*=1583), and 41% remained alive without ESKD at the end of the study period (*N*=4827). Of the total 12,217 patients, 1720 (14%) had kidney transplant, 1281 (10%) had dialysis, and 391 (3%) had both dialysis and kidney transplant during the study period. Most patients did not have records of dialysis or transplant (Table 1). Comparing the dialysis/transplant records in the VHA and those in USRDS revealed that VHA has records for most of the patients with ADPKD. For example, USRDS has kidney transplant records for 1792 (72 more patients that the records in VA CDW).

**Table 1:** Demographic characteristics of Veteran Affairs autosomal dominant polycystic kidney disease cohort

Characteristic	Value (*N*=12,217)
**Sex, *n* (%)**	
Male	11,426 (94)
Female	791 (6)
Age, yr, median (IQR)	63 (52–72)
**Age, yr, *n* (%)**	
<18	3 (0.02)
18–34	665 (6)
35–49	1741 (14)
50–64	4314 (35)
65–79	4285 (35)
80+	1209 (10)
**Self-reported race, *n* (%)**	
White	8088 (66)
Black or African American	2273 (19)
Asian	96 (0.8)
Native Hawaiian or other Pacific Islander	117 (1)
American Indian or Alaska Native	66 (0.5)
Unknown	1577 (13)
**Ethnicity, *n* (%)**	
Non-Hispanic or Latino	10,325 (84)
Hispanic or Latino	478 (4)
Unknown	1414 (12)
**ESKD/vital status, *n* (%) 11,752^a^**	
Alive non-ESKD	4827 (41)
Alive ESKD	1583 (14)
Deceased non-ESKD	3940 (33)
Deceased ESKD	1402 (12)
**Transplant/dialysis status, *n* (%)^b^**	
Kidney transplant	1720 (14)
Dialysis	1281 (10)
Both	391 (3)
Neither	9607 (79)

IQR, interquartile range.

a The number is smaller than the cohort size due to the exclusion of patients with missing vital status or without clean and meaningful eGFR values to confirm the ESKD status.

b The percentages do not sum to 100% due to 391 overlapping patients between dialysis and transplant and values rounding.

The prevalence of ADPKD in the VA population was calculated to be 0.05% (12,217/24 million total patient records). Age and sex distributions were similar across races (Table 2). The yearly new diagnosis (incidence) analysis showed that the proportion of black veterans steadily increased over the years from 12% to 22% by 2019 (Supplemental Table 2). The large number of new diagnosis in 2000 reflects the cumulated cases at the beginning of electronic record system. There is also a larger proportion of patients with missing/unknown race data in the first few years but decreased dramatically afterward.

**Table 2:** Age and sex distributions by race

Race	Age (Median, IQR)	Sex (% Male)
White	73 (65–83)	94
Black or African American	70 (61–78)	90
Asian	67 (56–73)	92
Native Hawaiian or other Pacific Islander	70 (60–81)	91
American Indian or Alaska Native	69 (60–75)	92
Unknown	82 (70–92)	95

IQR, interquartile range.

To characterize general ADPKD-associated comorbidities in the VA-ADPKD cohort, we extracted the data for the 29 commonly occurring chronic comorbidities used for the calculation of the Elixhauser comorbidity score. Consistent with known ADPKD manifestations, general comorbidities with higher prevalence in the VA ADPKD cohort (versus the general VA population^16,17^) included hypertension (89.0% versus 50%) and renal failure (67% versus 0.6%) (Table 3). Additional comorbidities with higher prevalence in the VA ADPKD cohort were depression (40% versus 10%), COPD (30% versus 6%), and diabetes (33% versus 22%). Obesity, while highly prevalent in this cohort, was less prevalent than in the general VA population (30% versus 40%). Low prevalence comorbidities in the VA-ADPKD cohort included HIV/AIDS (0.5%), chronic blood loss anemia (1%), lymphoma (2%), and paralysis (2%).

**Table 3:** Chronic comorbidities by race

Comorbidity	All Races, *N*=12,217 (%)^a^	Black Patients, *n*=2273 (%)	White Patients, *n*=8088 (%)
HIV/AIDS	59 (0.5)	29 (1)	23 (0.3)
Alcohol abuse	1310 (11)	373 (16)	832 (10)
Iron deficiency anemia	1738 (14)	502 (22)	1068 (13)
Rheum arthritis/collagen vascular diseases	483 (4)	104 (5)	321 (4)
Chronic blood loss anemia	158 (1)	51 (2)	89 (1)
Congestive heart failure	2609 (21)	596 (26)	1697 (21)
COPD	3684 (30)	685 (30)	2594 (32)
Coagulopathy	882 (7)	186 (8)	631 (8)
Depression	4908 (40)	1070 (47)	3371 (42)
Diabetes	3985 (33)	858 (38)	2629 (33)
Drug abuse	1086 (9)	379 (17)	625 (8)
Hypertension	10,873 (89)	2134 (94)	7214 (89)
Hypothyroidism	1568 (13)	205 (9)	1184 (15)
Liver disease	1496 (12)	367 (16)	996 (12)
Lymphoma	218 (2)	34 (2)	168 (2)
Fluid and electrolyte disorders	3615 (30)	901 (40)	2386 (30)
Metastatic cancer	421 (3)	94 (4)	278 (3)
Other neurological disorders	1082 (9)	262 (12)	721 (9)
Obesity	3601 (30)	741 (33)	2554 (32)
Paralysis	196 (2)	69 (3)	111 (1)
Peripheral vascular disorders	2711 (22)	545 (24)	1853 (23)
Psychoses	790 (7)	209 (9)	505 (6)
Pulmonary circulation disorders	532 (4)	145 (6)	352 (4)
Renal failure	8193 (67)	1765 (78)	5296 (66)
Solid tumor without metastasis	2666 (22)	620 (27)	1726 (21)
Peptic ulcer disease excluding bleeding	398 (3)	91 (4)	258 (3)
Valvular disease	1323 (11)	260 (11)	909 (11)
Weight loss	1096 (9)	310 (14)	686 (9)
Elixhauser index^b^	31 (17)	34 (21)	31 (16)
Intracranial aneurysm	269 (2)	68 (3)	171 (2)
Bronchiectasia	100 (0.8)	15 (0.7)	80 (1)
Intracranial bleeding	41 (0.3)	9 (0.4)	27 (0.3)
Cystic pancreas	610 (5)	120 (5)	417 (5)
Infertility	57 (0.5)	12 (0.5)	40 (0.5)
Nephrolithiasis	2000 (16)	338 (15)	1450 (18)
Pain	7457 (61)	1599 (70)	5074 (63)
Seminal vesical cysts	51 (0.4)	11 (0.4)	39 (0.5)

COPD, chronic obstructive pulmonary disease; IQR, interquartile range.

a All races group included races in addition to Black patients and white patients.

b Median (interquartile range).

To better understand the prevalence of additional comorbidities commonly associated with ADPKD, we extracted the relevant clinical data from the ADPKD cohort (Table 3). For example, in the VA-ADPKD cohort, the prevalence of diagnoses for nephrolithiasis is 16%, intracranial aneurysms 2%, intracranial bleeding 0.3%, bronchiectasia, 0.8%, seminal vesical cysts 0.4%, infertility 0.5%, and pain 61%. The suicide rate was 0.6%. We were not able to extract comparable data for the entire VA population for comparison's sake for all of these comorbidities.

The prevalence of all comorbidities was compared between Black/African American ADPKD patients and White patients. In general, although not all effects were statistically significant at the 0.05 level, Black/African American patients had higher rates of most comorbidities than White patients. Black/African American patients had Elixhauser scores an average of 2.19 points higher than White patients (95% confidence interval, 1.53 to 2.86). Notably, high effect sizes included HIV/AIDS (OR: 4.53 [2.62 to 7.85]), drug abuse (OR: 2.39 [2.08 to 2.74]), and paralysis (OR: 2.25 [1.66 to 3.05]) when comparing Black/African American patients with White patients (Table 3).

To benefit the future use of the VA-ADPKD cohort, we extracted 19 laboratory values that are potentially relevant to ADPKD prognosis (Table 4). The most common laboratory measurements included mean arterial pressure, body mass index/body surface area, potassium, carbon dioxide, glucose, and BUN. The average patient had these laboratory test results measured every 2–4 months. Least-often measured laboratory test results in this cohort included urine protein-to-creatinine ratio, urine albumin-to-creatinine ratio, and uric acid. Respectively, only 13%, 25%, and 54% of the cohort had a single measurement taken for these laboratory test results over the entire study period. For all laboratory test results studied, the median laboratory values fall into the “normal” range for all tests, supporting our data cleaning methods as being sufficient.

**Table 4:** Descriptive statistics for key laboratory values

Laboratory Test	No. of Patients with ≥1 Value	Laboratory Value, Median^a^ (IQR^b^)	No. of Laboratory Values per Person, Median (IQR)	Time between Laboratory Values (mo), Median (IQR)
Hemoglobin, g/dl	8,602	13.4 (12.0–14.7)	22 (9–47)	3.6 (0.7–7.1)
WBC count, 1000 cells/m^3^	8602	6.7 (5.7–8.0)	22 (9–49)	3.4 (0.9–6.9)
Potassium, mEq/L	11,342	4.3 (4.1–4.5)	28 (11–57)	3.1 (1.0–6.0)
Carbon dioxide, mEq/L	11,064	26.0 (24.0–28.0)	27 (11–56)	3.1 (1.0–6.1)
ALT/SGPT, IU/L	9661	21.0 (16.0–29.0)	15 (6–28)	5.9 (3.0–8.9)
AST/SGOT, IU/L	9524	21.0 (17.0–25.0)	15 (6–29)	5.8 (3.0–8.9)
Albumin, g/dl	11,059	3.9 (3.7–4.2)	18 (7–36)	4.4 (2.0,7.3)
Alk. phosphatase, IU/L	9475	74.0 (61.0–90.0)	15 (6–29)	5.7 (2.8–8.8)
Uric acid, mg/dl	6616	6.7 (5.6–7.8)	4 (2–9)	6.6 (3.6–13.8)
Protein, g/dl	10,497	7.1 (6.7–7.4)	15 (6–30)	5.2 (2.4–8.3)
Glucose, mg/dl	11,272	101.0 (94.0–113.0)	27 (11–56)	3.1 (1.0–7.2)
Phosphorus, mg/dl	7745	3.5 (3.1–4.0)	9 (3–25)	3.4 (0.9–7.2)
Calcium, mg/dl	11,172	9.3 (9.0–9.5)	25 (9–51)	3.2 (1.0–6.1)
BUN, mg/dl	11,277	24.0 (17.05–35.0)	28 (11–58)	2.9 (0.9–6.0)
Body surface area, m^2^	12,139	2.1 (1.9–2.3)	36 (16–73)	1.9 (1.0–4.3)
BMI, kg/m^2^	12,139	28.2 (25.1–31.9)	36 (16–73)	1.9 (1.0–4.3)
MAP, mm Hg	12,149	96.7 (91.7–101.3)	65 (24–153)	0.6 (0.03–1.9)
Urine ACR, mg/g	3106	20.5 (4.5–91.5)	3 (2–8)	12.3 (3.5–15.0)
Urine PCR, mg/g	1618	0.8 (0.1–128.0)	3 (1–6)	7.1 (4.4–13.1)

ALT, alanine aminotransferase; AST, aspartate aminotransferase; BMI, body mass index; IQR, interquartile range; MAP, mean arterial pressure; SGOT, serum glutamate-oxaloacetate transaminase; SGPT, serum glutamate-pyruvate transaminase; Urine ACR, urine albumin to creatinine ratio; Urine PCR, urine protein to creatinine ratio; WBC, white blood cell.

a Median laboratory value was taken at the patient level first and the medians and interquartile range were calculated across the whole cohort.

b Interquartile range or 25th and 75th percentile.

**Figure 1:**
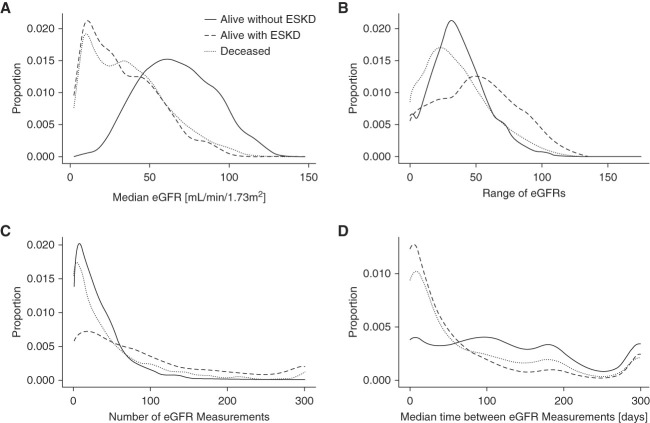
**Patient-level descriptive statistics of eGFR measurements in the VA-ADPKD cohort.** Proportions were approximated with a density function. The distribution of median patient-level eGFR values among alive, non-ESKD patients was higher than the distributions among alive ESKD patients and patients who died during the study period (A). The range of eGFR values of alive patients with ESKD tends to be larger than those of deceased and alive without ESKD (B). As expected, the total number of eGFR values in the VHA system over 20 years is the largest for patients who are alive but have reached ESKD (C), and the eGFR measurements are sparser among patients who are alive and have not reached ESKD (D). ADPKD, autosomal dominant polycystic kidney disease; VA, Veteran Affairs; VHA, Veterans Health Administration.

The patients with ADPKD in our study cohort (*n*=12,217) were heterogeneous in terms of disease progression phase. Therefore, we divided the patient cohort into three groups, alive without reaching ESKD, alive but with ESKD, and deceased. The eGFR is the key indicator of renal function in patients with ADPKD. We summarized the eGFR measurements of the patients in the three groups in the VA-ADPKD cohort using the median and range of eGFR values (Figure 1, A and B), the number of measurements (Figure 1C), and the time distance between eGFR measurements (Figure 1D). The median patient-level eGFR values among alive, non-ESKD patients (overall median: 65.95; IQR: 49.27–84.15) were generally higher than those among alive ESKD patients (28.49 [14.33–48.41]) and patients who died during the study period (33.62 [16.17–51.99]). Furthermore, the median eGFR values were very similar between the deceased and alive with ESKD groups (Figure 1A). More than half of the alive ADPKD patients without ESKD had a median eGFR 65.95 and above. By contrast, only 9% of alive ADPKD patients who have reached ESKD and 13% of deceased ADPKD patients had a median eGFR 65.95 and above. The range of within-patient eGFR values differ among the three groups of patients (Figure 1B). The alive ESKD patients had the highest range (maximum–minimum) of eGFR measurements over the study period with a median range of 49.75 (versus 34.91 for the alive without ESKD group and 31.80 for the deceased group).

Patient-level number of eGFR measurements available in the VA system over the study period varies substantially. The majority of alive non-ESKD patients and deceased patients had between 0 and 50 eGFR measurements over the study period, whereas alive ESKD patients tended to have more measurements (Figure 1C). In addition, alive ADPKD patients without ESKD tended to have more time between measurements than the other two groups (Figure 1D). The median time between eGFR measurements was 115.89 days for ADPKD patients without ESKD (versus 28.02 for alive with ESKD group and 39.47 for the deceased group).

## Discussion

To our knowledge, the VA-ADPKD cohort (12,217 patients with ADPKD) is currently the largest ADPKD patient cohort with sufficient clinical data to support potential predictive modeling of key ADPKD outcomes (*e.g*., eGFR trajectories and CKD stage end points). It also has one of the highest representations of Black/African American patients (19%) among currently available ADPKD datasets, unlike most clinical ADPKD datasets where this population is typically significantly underrepresented.

The disproportionate representation of Black/African American patients in the VA-ADPKD cohort (19% versus 12% in the overall VA patient population) is consistent with reports from the KPSC health system EHR database (12% versus 7% in the overall Kaiser population). Both of these population-wide studies suggest that Black/African American patients are more frequently affected by ADPKD compared with all other races. Further research is needed to validate this finding due to the risk of sampling bias.

The remaining demographic characteristics of the VA-ADPKD cohort mostly resemble the entire VA patient population (*e.g*., age and sex and ethnicity distributions^16^). For example, this ADPKD cohort has a somewhat higher diabetes prevalence (33%) compared with the general VA patient population (22%).^16^ However, the obesity rate (30%) in this ADPKD cohort was lower than the estimated rate of veterans nationwide (41%).^18^ We also observed higher rates of depression (40% versus 10%),^19^ congestive heart failure (21% versus 1%),^16^ COPD (30% versus 6%), and hypertension (89% versus 50%)^16^ in the ADPKD cohort compared with the general VA population. While higher rates of hypertension,^20^ depression,^21^ and cardiovascular complications^22^ among patients with ADPKD have been previously established, the notable increase of COPD among ADPKD veterans has not been, to our knowledge, previously reported.

Most laboratory data extracted from the VA CDW were available for most cohort, with the exception of urine protein-to-creatinine ratio and urine albumin-to-creatinine ratio since these laboratory measures are tested less frequently. Data quality checks revealed generally normal ranges of laboratory values, validating our cleaning measures. Therefore, our longitudinal data collected over 20 years should represent a robust resource for both characterization of various ADPKD manifestations, their progression, as well as development of statistical models to predict these outcomes.

Limitations of this study include the use of ICD codes for phenotyping both ADPKD and comorbidities. Other more advanced phenotyping methods on the basis of image analysis can be more accurate, although phenotyping ADPKD *via* ICD codes has been shown to be quite accurate in previous work by Kalot *et al.*^23^ The authors found that ICD codes yielded an 82% specificity for those who did not attend nephrology clinics and an 84% specificity for those who did. The sensitivity values were 97% and 99%, respectively. In addition, data on kidney size and cortical thickness were not available for this cohort which limits the identifiability of patients with ADPKD patients, particularly from autosomal dominant tubulointerstitial kidney disease and acquired cystic kidney disease. Another limitation of this cohort is the lack of non-VA data available for these patients, which would help provide a more complete picture of the patients' health over time. In addition, the veteran population is predominantly male, older, and of lower socioeconomic status than the general population, making results from our cohort less generalizable. Any future prognostic modeling on the basis of this cohort will predominantly apply to those subgroups.

In summary, we have established the largest existing ADPKD patient cohort using national US Veteran EHR data. In future work, we plan to use the laboratory and comorbidity data available for this cohort to improve the existing knowledge around ADPKD, its progression, and its complications. Finally, we will also use this cohort of veterans to further validate the recently observed phenomenon of Black/African American patients having disproportionately high rates of ADPKD.

## Supplementary Material

## Data Availability

All data is included in the manuscript and/or supporting information.

## Supplemental Material

Supplemental Table 1. Plausible ranges for laboratory measurements.

Supplemental Table 2. Number of newly diagnosed cases stratified by race in each year for the 20-year study period.
