# Supplementary material for: US Veterans Administration Autosomal Dominant Polycystic Kidney Disease Cohort: Demographic, Comorbidity, and Key Laboratory Data Characteristics
Source: Kidney360. 2024 Mar 1;5(4):529–37. doi: 10.34067/KID.0000000000000405 (PMC11093548; doi:10.34067/KID.0000000000000405)
Supplement: Supplementary file 1 [file kidney360-5-529-s001.pdf]

Suppl. Table 1. Plausible Ranges for Lab Measurements

Lab Test	Range of Plausible Values (Implausible excluded)
Hemoglobin (g/dL)	[5, 25]
White Blood Cell Count (1000 cells/ m^3)	[1, 100]
Potassium (mEq/L)	[1.5, 12]
Carbon Dioxide (mEq/L)	[5, 65]
ALT/SGPT (IU/L)	[0, 8000]
AST/SGOT (IU/L)	[0, 8000]
Albumin (g/dL)	[0, 10]
Alkaline Phosphatase (IU/L)	[5, 1000]
Uric Acid (mg/dL)	[0.5, 20]
Protein (g/dL)	[4, 20]
Glucose (mg/dL)	(0, 2000]
Phosphorus (mg/dL)	[0, 15]
Calcium (mg/dL)	[4, 15]
Blood Urea Nitrogen (mg/dL)	No restrictions
Height (in.)	[50, 85]
Weight (lbs.)	[70, 600]
Systolic Blood Pressure (mmHg)	>0
Diastolic Blood Pressure	(0, 160]
Urine Albumin Creatinine Ratio	No restrictions
Urine Protein Creatinine Ratio	No restrictions

Suppl. Table 2. Number of Newly Diagnosed Cases Stratified by Race in Each Year for the 20-year Study Period

Year	White (%)	Black or African American (%)	American Indian or Alaska Native (%)	Asian (%)	Native Hawaiian or Other Pacific Islander (%)	Unknown (%)	Missing (%)	Total
2000	582 (53%)	136 (12%)	4 (0.4%)	6 (0.5%)	12 (1.1%)	359 (33%)	0 (0)	1099
2001	384 (56%)	92 (13%)	7 (1%)	3 (0.4%)	4 (0.6%)	198 (29%)	0 (0)	688
2002	393 (60%)	102 (16%)	0 (0)	2 (0.3%)	5 (0.8%)	149 (23%)	0 (0)	651
2003	466 (65%)	109 (15%)	3 (0.4%)	6 (0.8%)	5 (0.7%)	128 (18%)	0 (0)	717
2004	434 (66%)	108 (17%)	5 (0.8%)	4 (0.6%)	7 (1.1%)	96 (15%)	0 (0)	654
2005	425 (71%)	111 (18%)	3 (0.5%)	0 (0)	4 (0.7%)	59 (10%)	0 (0)	602
2006	437 (73%)	85 (14%)	3 (0.5%)	4 (0.7%)	7 (1.2%)	62 (10%)	0 (0)	598
2007	406 (71%)	100 (17%)	0 (0)	9 (1.6%)	3 (0.5%)	54 (9%)	1 (0.2%)	573
2008	405 (69%)	109 (19%)	5 (0.9%)	6 (1%)	8 (1.4%)	51 (9%)	0 (0)	584
2009	401 (69%)	119 (21%)	4 (0.7%)	5 (0.9%)	8 (1.4%)	42 (7%)	0 (0)	579
2010	417 (70%)	124 (21%)	3 (0.5%)	5 (0.8%)	2 (0.3%)	46 (8%)	0 (0)	597
2011	447 (74%)	117 (19%)	2 (0.3%)	1 (0.2%)	7 (1.2%)	31 (5%)	0 (0)	605
2012	464 (69%)	158 (24%)	4 (0.6%)	3 (0.4%)	7 (1%)	32 (5%)	0 (0)	668
2013	487 (71%)	138 (20%)	7 (1%)	2 (0.3%)	8 (1.2%)	46 (7%)	0 (0)	688
2014	451 (68%)	151 (23%)	5 (0.8%)	13 (2%)	8 (1.2%)	34 (5%)	0 (0)	662
2015	420 (68%)	158 (26%)	4 (0.6%)	7 (1.1%)	10 (1.6%)	20 (3%)	0 (0)	619
2016	276 (69%)	87 (22%)	1 (0.3%)	4 (1%)	6 (1.5%)	24 (6%)	0 (0)	398
2017	288 (70%)	89 (22%)	3 (0.7%)	7 (1.7%)	2 (0.5%)	23 (6%)	0 (0)	412
2018	245 (68%)	75 (21%)	1 (0.3%)	3 (0.8%)	2 (0.6%)	29 (8%)	6 (1.7%)	361
2019	243 (57%)	94 (22%)	2 (0.5%)	6 (1.4%)	2 (0.5%)	37 (9%)	39 (9.2%)	423
2020	17 (44%)	11 (28%)	0 (0)	0 (0)	0 (0)	7 (18%)	4 (10.3%)	39
Total	8088 (66%)	2273 (19%)	66 (0.5%)	96 (0.8%)	117 (1%)	1527 (12%)	50 (0. 4%)	12217

Note: Proportion of Black/African-American veteran increased over the years from 12% to 22%.
